# scSpatialSIM: a simulator of spatial single-cell molecular data

**DOI:** 10.1016/j.softx.2025.102223

**Published:** 2025-06-06

**Authors:** Alex C. Soupir, Julia Wrobel, Jordan H. Creed, Oscar E. Ospina, Christopher M. Wilson, Brandon J. Manley, Lauren C. Peres, Brooke L. Fridley

**Affiliations:** a Department of Biostatistics and Bioinformatics, Moffitt Cancer Center, Tampa, FL, United States; b Department of Genitourinary Oncology, Moffitt Cancer Center, Tampa, FL, United States; c Department of Biostatistics, Emory University, Atlanta, GA, United States; d Department of Cancer Epidemiology, Moffitt Cancer Center, Tampa, FL, United States; e Division of Health Services and Outcomes Research, Children’s Mercy, Kansas City, MO, United States

**Keywords:** Spatial molecular data, Cell clustering, Cell co-localization, Spatial statistics benchmarking, Spatial single-cell simulations

## Abstract

The increasing use of spatial molecular technologies such as multiplex immunofluorescence (mIF) and spatial transcriptomics (SRT) has driven the need for robust statistical methods to analyze the spatial architecture of tissues. However, a lack of consensus on “gold standard” approaches present challenges for benchmarking and comparison. To address this gap, we developed “scSpatialSIM”, an *R* package for simulating biologically realistic spatial single-cell molecular data. “scSpatialSIM” enables users to efficiently simulate single-cell spatial patterns without requiring reference datasets, incorporating features such as cell clustering, cell co-localization, tissue compartments, and tissue holes. Additionally, the package supports simulation of both categorical data (e.g., cell phenotypes) and continuous values (e.g., protein expression or gene expression), and integrates with other *R* packages for downstream spatial analyses. To demonstrate its utility, we applied “scSpatialSIM” to benchmark univariate point pattern summary functions, including Ripley’s K(*r*), nearest neighbor G(*r*), and pair correlation g (*r*), across simulated scenarios. The results showed that Ripley’s K(*r*) consistently detected clustering across multiple radii, outperforming other methods in sensitivity and robustness. While scSpatialSIM is limited to simulating cell clustering and co-localization rather than broader tissue-level sub-domains, it provides a flexible and scalable framework for generating diverse spatial data. The development of scSpatialSIM facilitates comparative evaluation of spatial statistics and enables researchers to explore hypothetical scenarios at scale, advancing the development of novel methods to characterize the spatial organization of tissues. By providing a platform for spatial simulation, scSpatialSIM supports innovation in spatial molecular research and fosters new insights into tissue architecture and cellular interactions.

## Metadata

**Table:** 

Nr	Code metadata description	Metadata
C1	Current code version	*V0.1.3.4*
C2	Permanent link to code/repository used for this code version	https://cran.r-project.org/web/packages/scSpatialSIM/; https://github.com/FridleyLab/scSpatialSIM/
C3	Permanent link to reproducible capsule	
C4	Legal code license	*MIT*
C5	Code versioning system used	*Git, CRAN*
C6	Software code languages, tools and services used	R
C7	Compilation requirements, operating environments and dependencies	R (≥4.0)
C8	If available, link to developer documentation/manual	https://fridleylab.github.io/scSpatialSIM/index.html
C9	Support email for questions	Fridley.Lab@moffitt.org

## 1. Motivation and significance

Studying the spatial contexture of cells in biological tissues is increasing in popularity among biomedical researchers. New technologies allow the profiling and phenotyping of cells in tissue microarrays (TMAs) and whole tissue slides to understand the spatial architecture of tissues, such as the tumor microenvironment (TME), and the distributions of cells in 2-dimensional space. The ability to characterize the spatial context of cells has allowed for further exploration of the correlation between the tissue architecture and patient outcomes, such as overall survival or response to therapies [1–3]. In contrast to studying only the cell abundance or density, the spatial architecture of cell types and their level of aggregation or dispersion can provide more details about the organization of complex tissues and answer important questions about cell-cell communication and their associations [3,4].

Single-cell protein imaging platforms like multiplex immunofluorescence (mIF) or immunohistochemistry [5], have been successfully used to profile cell types in tissues, as well as spatially characterize the immune microenvironment of several malignancies [3,6]. In addition to single-cell protein data, spatially resolved transcriptomics (SRT) has recently gained traction in biological research. SRT experiments can range from studies involving imaging or sequencing regions of tissue (GeoMx [7], Nanostring, Seattle, WA) to equally spaced grids with unique molecular identifiers and spatial barcodes containing between 5 and 10 cells (Visium [8], 10x Genomics, Pleasanton, CA) to sub-cellular transcript locations (CosMx Spatial Molecular Imaging [9], Nanostring, Seattle, WA; Xenium [10], 10x Genomics). These spatial molecular technologies require complex statistical and bioinformatic approaches to summarize the spatial characteristics of the tissues. Statistical methods from the field of ecology have been used to assess the level of clustering of cell types in tissues, but there is a lack of consensus on which method is the “gold standard”. Along with lacking a ‘gold standard’, methods for analyzing the spatial contexture of cells in both spatial proteomics and SRT continue to be developed rapidly yet lack the ways for which to objectively evaluation them against existing methods.

**Fig. 1.**
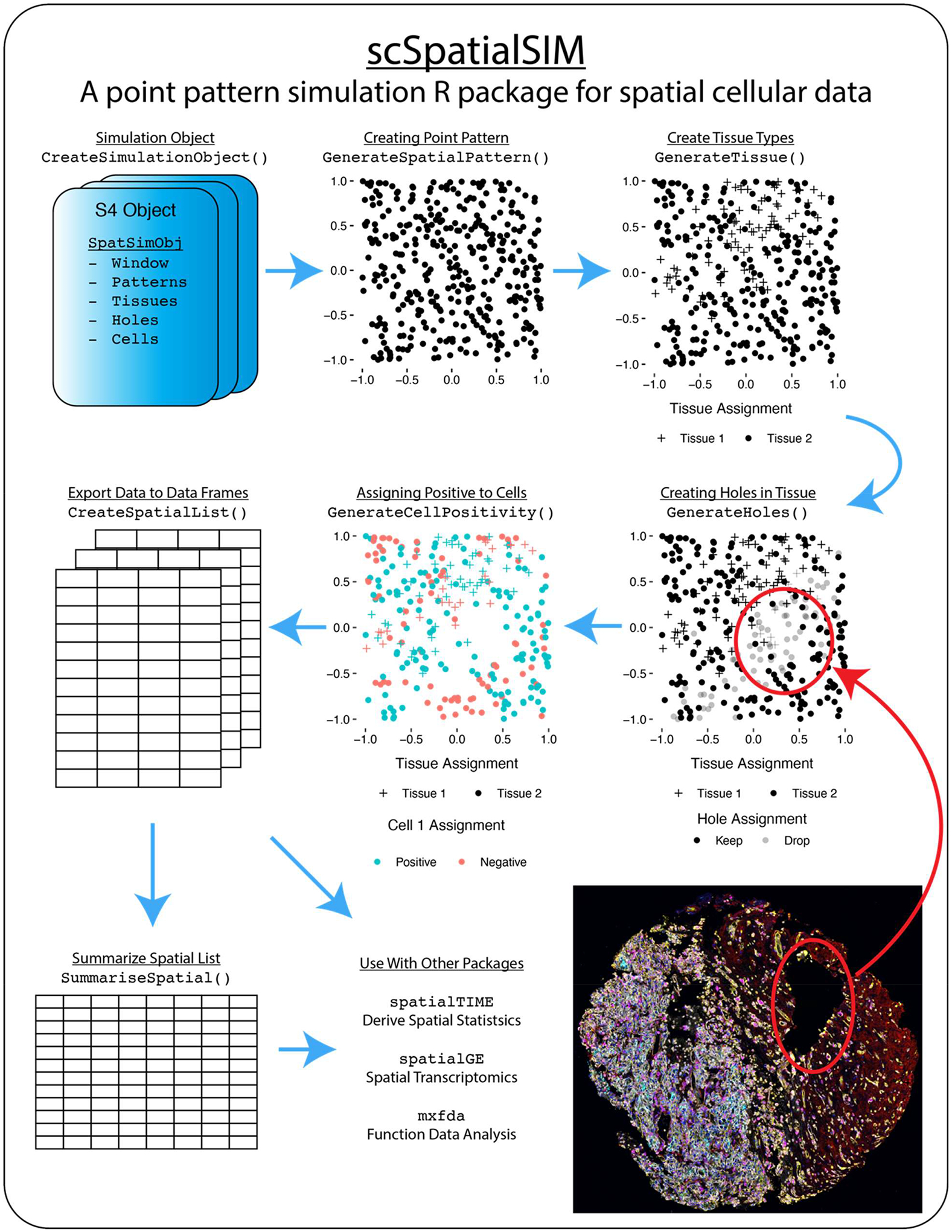
Workflow used by “scSpatialSIM” to generate point patterns, tissue domains, tissue holes, and cell positivity. These data can then be exported and summarized for use by other packages.

To bridge this gap in the literature, we have developed an *R* package, “scSpatialSIM”, that allows for fine-tuned simulation of spatial single-cell molecular data which can be exported in formats that are common for the field (i.e., HALO from Indica Labs for digital pathology; “scSpatialSIM” workflow is displayed in Fig. 1). This package can be used to compare available statistical methods for measuring clustering and co-localization of single-cell spatial data as well as benchmark new methods as they are developed. While packages like “spaSim” [11], “scCube” [12], “scDesign3” [13], and “SRTsim” [14] exist to simulate cell-level data to mimic tissue architectures and domains, “scSpatialSIM” can simulate cell phenotypes (categorical) and continuous data (e.g., mIF intensity data, SRT gene expression data) depending on user input and parameter settings without requiring training data. Table 1 display comparisons between these simulation methods. The distributions of protein or gene expression for positive and negative cells can be finely adjusted to assess spatial clustering and co-localization method sensitivity.

## 2. Software description

“scSpatialSIM” is an *R* package designed to simulate spatial single-cell molecular data, offering researchers a flexible and efficient platform for creating biologically realistic spatial data to benchmark clustering methods. The package supports installation on R4.0.0 or later and is available from GitHub and CRAN. Many of the functions from “scSpatialSIM” support piping (i.e., using the magrittr %>% function) which allows function calls to be chained together to develop whole simulation pipelines from start to finish. Once scenarios have been simulated, “scSpatialSIM” can export the results as tabular data (i.e., Indica Labs’ HALO) for use in external tools to assess power of spatial clustering methods. Additionally, we have created vignettes (https://fridleylab.github.io/scSpatialSIM) that demonstrate how to use “scSpatialSIM” as well as how the outputs of “scSpatialSIM” can be input into other R packages to derive spatial statistics, such as with the R package “spatialTIME” [15].

### 2.1. Software architecture

**Fig. 2.**
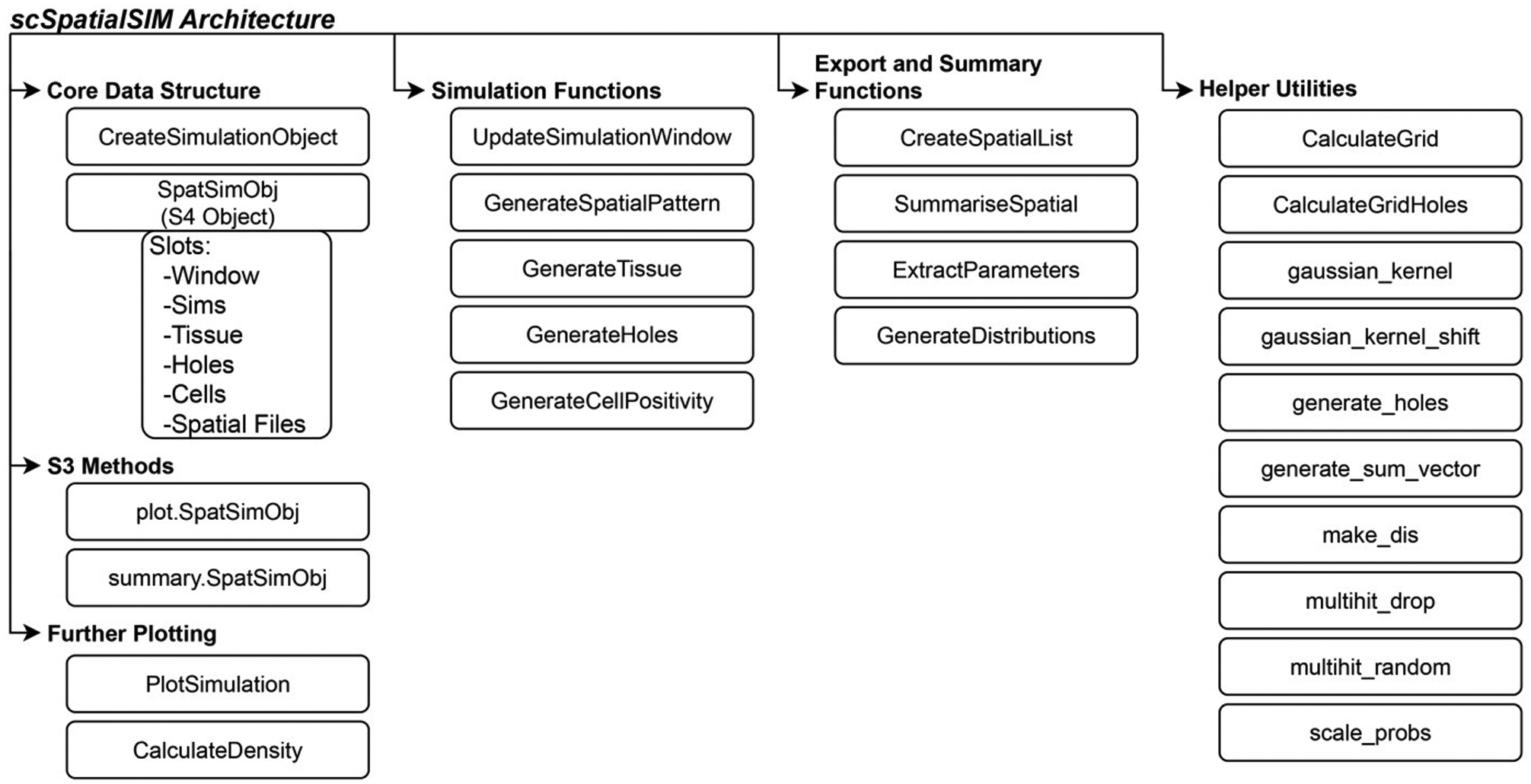
Structure of scSpatialSIM package.

“scSpatialSIM” uses an S4 object (class SpatSimObj) which serves as storage for all the parameters and results performed with “scSpatialSIM”. Our goal was to provide a collection of methods that allow for the creation of realistic simulations of cell types within tissue samples in a stepwise manner: 1) point patterns, 2) tissue regions or domains, 3) holes/non-cellular areas, and 4) cell phenotypes. Fig. 2 shows the architecture of “scSpatialSIM”. The SpatSimObj also stores the simulation space (class òwin` from “spatstat” [16]), the number of independent simulations to perform, and the number of cell types to simulate.

### 2.2. Software functionalities

The simulation functionality of “scSpatialSIM” is handled largely through 5 functions from the creation of the SpatSimObj object to generating cell positivity from the probability surfaces:

1. CreateSimulationObject(): Creates the S4 object that maintains information about the simulation scenario from the simulation window (“spatstat” object class owin), the number of simulations, and the probability surface layers with their respective parameters. Additionally, the number of cell types wanting to be simulated is stored here (one cell type for benchmarking clustering, or more than one for benchmarking colocalization)
2. GenerateSpatialPattern(): Generates spatial point patterns using “spatstat” rpoispp(). This point pattern represents the underlying point pattern of the whole sample.
3. GenerateTissue(): Generates a probability surface based on input parameters for regions that will be “Tissue 1″, with areas of low probability being assigned “Tissue 2”.
4. GenerateHoles(): Generates a probability surface based on input parameters for which points in the point pattern have a feature of ‘hole’ for evaluating spatial metrics ability to compensate for violations of stationarity.
5. GenerateCellPositivity(): Generates probability surfaces based on input parameters and number of cell types set in the SpatSimObj. This results in cells being labelled as positive and negative. This function also allows for shifting of the probability surfaces between multiple cell types

Further, GenerateDistributions() can be used to generate distributions on positive and negative cells (i.e., protein abundance, gene expression, etc.) to assess power of spatial functions to pick up varying degrees in shifts spatially between cells types.

During the simulation process, there is the ability to calculate probabilities at differing spatial resolutions in functions used to calculate probability surfaces. If probabilities at the different resolutions are not initially calculated (desired if simulating many samples in a scenario), CalculateDensity() is able to calculate them at a later step for visualization. PlotSimulation() allows for plotting of these probabilities as a heatmap to visualize the probability surface used for cell assignments. It is also able to plot the whole core after cells have been assigned to the tissues, holes, and cell types. A basic point pattern is able to be visualized using the S3 plot method from ‘spatsat’ [16].

To provide the data in formats that are typical outputs from software suits like Indica Labs’ HALO and input formats for tools like “spatialTIME” or “spatstat”, we’ve included CreateSpatialList() that returns a list of data frames, one for each sample (each row a cell). Often these data frames are also summarized at the sample or image level, with each row containing number of cells and cell abundance for a sample, which can be calculated with SummariseSpatial().

## 3. Illustrative examples

To demonstrate the utility of “scSpatialSIM”, we will use the example of benchmarking the univariate spatial summary functions Ripley’s K, Nearest Neighbor G, and Pair Correlation g with 4 simulation scenarios that differ in abundance and size of clusters. Benchmarking of the spatial summary functions can have two separate components: 1) which spatial summary function is able to identify the most samples in a scenario with significant clustering, and 2) how many radii does each spatial summary function identify 100 % of samples with significant clustering. Significant clustering is assessed through a permutation approach to determine the empirical distribution of randomness.

### 3.1. scSpatialSIM example code


#Create simulation object with 1000 samples in a 2×2 window, centered at 0
*R*> obj = CreateSimulationObject(sims = 1000,
+ window = spatstat.geom::owin(c(−1, 1), c(−1, 1)))
#generate spatial point pattern with a point (cell) intensity of 250 per unit area
*R*> obj = GenerateSpatialPattern(obj,
+ lambda = 250)
#generate probability surface and assign points (cells) to holes, with default parameters
*R*> obj = GenerateHoles(obj, density_heatmap = FALSE,
+ cores = 5, use_window = TRUE)
#generate probability surface and assign points (cells) to tissues, with default parameters
*R*> obj = GenerateTissue(obj, density_heatmap = FALSE,
+ cores = 5, use_window = TRUE)
#generate positivity for cells – since univariate shift won’t be used
#simulating 5 clusters (*k* = 5)
#tight clusters (small sdmin and sdmax)
#high abundance (probs between 0.01 and 0.75 for probability surface scaling)
*R*> obj = GenerateCellPositivity(obj,
+ *k* = 5, sdmin = 0.1, sdmax = 0.3,
+ probs = *c*(0.01, 0.75), use_window = TRUE)
#extract the new spatial samples and summarise at sample level
*R*> spat_list = CreateSpatialList(obj)
*R*> sum_data = SummariseSpatial(spatlist, ‘Cell 1 Assignment’)


### 3.2. Different simulation scenarios

Running “scSpatialSIM” for 4 different scenarios provides different degrees of clustering to test the spatial summary functions under. Table 2 shows the different scenarios simulated with varying clusters size (Cluster Variation) and abundance (Cell Abundance). These parameters can be gathered from biological samples to replicate real-world tissues.

### 3.3. Assessing spatial summary functions

**Fig. 3.**
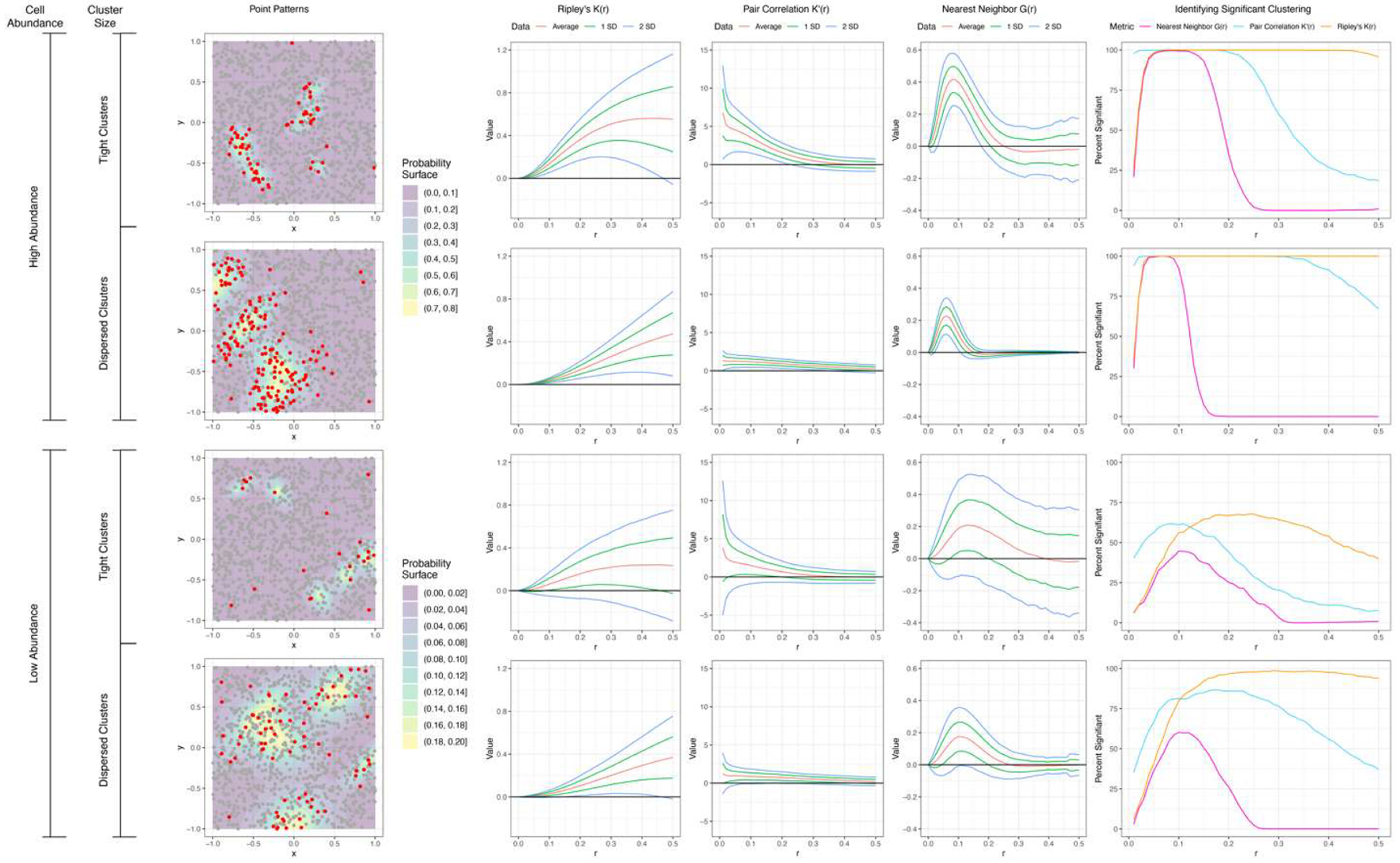
Spatial summary functions calculated for 4 simulation scenarios. Point patterns simulated with “scSpatialSIM” to emulate high/low kernel density (high/low abundance) and high/low kernel variance (large/small clusters). Results of Ripley’s K(*r*), nearest neighbor G(*r*), and pair correlation g(*r*) for radii from 0 to 0.5. Proportion of samples with significantly higher observed clustering than expected for each spatial summary function.

Using these simulated scenarios, we applied spatial summary functions within the “spatialTIME” package R package (Ripley’s K, Nearest Neighbor G, and Pair Correlation g). A total of 100 permutations were used to estimate the deviation of real Cell 1 Assignment locations from the empirical distribution of randomness, and each function was estimated at radii from 0 to 0.5 at increments of 0.01. Fig. 3 shows example sample plots for the 4 scenarios as well as Degree of Clustering Permutation from “spatialTIME” averaged over all samples (with mean and standard deviations).

The results of the “scSpatialSIM” simulated data show that Ripley’s K identifies significant cell clustering across more radii than Nearest Neighbor G and Pair Correlation g (Table 3 – Most Significant). Scenarios with high abundance (Scenario 1 and 2) also showed more radii with all simulated samples with significant clustering for Ripley’s K and Pair Correlation g. Nearest Neighbor G did not identify more samples with significant clustering at any radii, and only identified all simulated samples having significant clustering at a single radius.

## 4. Impact

Spatial profiling is constantly increasing in usage in biological sciences [17], and with it new spatial methods are being developed in attempt to better capture tissue features. However, there is little means of comparing methods objectively and under which environments the methods are best suited. “scSpatialSIM” is an easy and fast software that allows for the fine-tuned simulation of biologically-informed clustering without the requirement for training data. With “scSpatialSIM”, researchers are now able to quickly benchmark spatial clustering methods at scale, including those meant for assessing spatial autocorrelation (demonstrated in vignette “Adding Continuous Cell Attributes”) and even impacts that violations of stationarity may result in (like necrotic tissue or tissue folds during sectioning). Previously, we have showed it’s utility in benchmarking bivariate spatial summary functions assessing immune cell colocalization in ovarian and lung cancer tumors [18]. Though, while the software focuses on single-cell molecular data it can directly be used as demonstrated here in other fields such as ecology.

“scSpatialSIM” is meant to simulate single cell clustering, or two or more cell colocalization. While it does lack the ability to simulate more complex tissue domains or regions that may be important for benchmarking methods designed to identify them, “scSpatialSIM” excels at its primary goal: providing a robust and scalable platform for benchmarking spatial statistics. With this software, researchers will be better able to benchmark new spatial clustering methods and select those that best answer their research questions.

## 5. Conclusions

“scSpatialSIM” is an important software for advancing spatial biology and method development. With its ability to simulate different clustering scenarios at scale, it will help identify correct methods to accurately answer biological questions. Further, since “scSpatialSIM” exports data in formats that are easily used in other packages that assess spatial clustering, it streamlines the process of benchmarking.

## Figures and Tables

**Table 1:** Comparison between scSpatialSIM and existing tools for spatial single-cell simulation.

	scDesign3	spaSim	SRTsim	scCube	scSpatialSIM
Language	R package	R package	R package	Python package	R package
Data Modalities	Continuous data (e.g., cell trajectories, spot-level transcriptomics, epigenomics)	Categorical data (e.g., phenotypes)	Continuous data (e.g., gene expression counts)	Continuous data (e.g., gene expression)	Categorical (e.g. cell types) and continuous (e.g. protein/gene expression) for spatial data only
Reference-Based?	Yes	No	Yes and No	Yes and No	No
Input Requirements	Matrices: cell-by-feature, cell-by-state, cell-by-design	User-defined parameters in functions	Optional reference dataset or user-defined spatial structure	Can use pretrained models (~300) or user-provided data and annotations	User-defined parameters; no training required (parameters may be from real data)
Simulation Focus	Simulating spatial transcriptomic clusters, pseudotime, and batch effects	Simulating colocalization	Simulating spatial transcriptomics expression patterns	Simulating expression and spatial variability across many settings	Simulating clustering, colocalization, and expression gradients
Hypothesis Testing	Can simulate null/alternative models	Can be used for benchmarking spatial metrics	Can be used to benchmark spatial clustering, spatial differential expression, and cell-cell communication methods	Can be used to benchmarking spot deconvolution, gene imputation, and spatial clustering methods	Can be used to benchmarking spatial clustering and spatial autocorrelation methods

**Table 2:** Parameters used in “scSpatialSIM” to generate 4 simulation scenarios.

Study	Point Pattern Intensity	Number of Clusters	Cluster Variation	Cell Abundance
Scenario 1	250	5	0.1 – 0.3	0.01 – 0.75
Scenario 2			0.2 – 0.4	
Scenario 3			0.1 – 0.3	0.01 – 0.2
Scenario 4			0.2 – 0.4	

**Table 3:** For each spatial summary function, the number of radii (out of 50 assessed) which they had identified significant clustering in the most samples (“Most Significant”) across the 4 simulation scenarios. Additionally for each spatial summary function, the number of radii (out of 50 assessed) where 100 % of samples were identified as significantly clustered (“All Sig.”) across the 4 simulation scenarios. The simulation scenarios are: Scenario 1 – high abundance in tight clusters; Scenario 2 – high abundance in dispersed clusters; Scenario 3 – low abundance in tight clusters; Scenario 4 – low abundance in dispersed clusters.

	Ripley’s K(*r*)		Pair Correlation g(*r*)		Nearest Neighbor G(*r*)	
Study	Most Significant	All Significant	Most Significant	All Significant	Most Significant	All Significant
Scenario 1	36	24	9	8	0	0
Scenario 2	23	46	4	25	0	1
Scenario 3	38	0	13	0	0	0
Scenario 4	40	0	10	0	0	0
